# Multivariate Analysis Identifies Eight Novel Loci Associated with Meat Productivity Traits in Sheep

**DOI:** 10.3390/genes12030367

**Published:** 2021-03-04

**Authors:** Alexander S. Zlobin, Pavel S. Nikulin, Natalia A. Volkova, Natalia A. Zinovieva, Baylar S. Iolchiev, Vugar A. Bagirov, Pavel M. Borodin, Tatiana I. Aksenovich, Yakov A. Tsepilov

**Affiliations:** 1 Kurchatov Genomics Center of IC&G, Siberian Branch of the Russian Academy of Sciences, 630090 Novosibirsk, Russia; zlobin@bionet.nsc.ru; 2 Institute of Cytology and Genetics, Siberian Branch of the Russian Academy of Sciences, 630090 Novosibirsk, Russia; leptoncloak@gmail.com (P.S.N.); borodin@bionet.nsc.ru (P.M.B.); aks@bionet.nsc.ru (T.I.A.); 3 L.K. Ernst Federal Research Center for Animal Husbandry, Dubrovitsy, 142132 Moscow Region, Russia; natavolkova@inbox.ru (N.A.V.); n_zinovieva@mail.ru (N.A.Z.); baylar2@mail.ru (B.S.I.); vugarbagirov@mail.ru (V.A.B.); 4 Department of Natural Science, Novosibirsk State University, 630090 Novosibirsk, Russia

**Keywords:** sheep, meat productivity, quantitative genetics, multivariate analysis, GWAS

## Abstract

Despite their economic value, sheep remain relatively poorly studied animals in terms of the number of known loci and genes associated with commercially important traits. This gap in our knowledge can be filled in by performing new genome-wide association studies (GWAS) or by re-analyzing previously documented data using novel powerful statistical methods. This study is focused on the search for new loci associated with meat productivity and carcass traits in sheep. With a multivariate approach applied to publicly available GWAS results, we identified eight novel loci associated with the meat productivity and carcass traits in sheep. Using an in silico follow-up approach, we prioritized 13 genes in these loci. One of eight novel loci near the *FAM3C* and *WNT16* genes has been replicated in an independent sample of Russian sheep populations (*N* = 108). The novel loci were added to our regularly updated database increasing the number of known loci to more than 140.

## 1. Introduction

Sheep meat is the fourth economically most important type of meat product [1]. Identification of quantitative trait loci (QTLs) and candidate genes that affect growth rate, carcass, and meat productivity traits is a prerequisite for the marker-assisted selection of these traits [2]. Genome-wide association studies (GWAS) are a modern, agnostic, data-driven method for identifying QTLs associated with different traits. We previously developed a comprehensive database of QTLs associated with growth and meat traits in sheep based on the available published GWAS and gene-candidate studies [3]. It is regularly updated with newly discovered loci, but only a few of them have been confirmed in independent studies or validated over the past seven years. Therefore, good quality annotation data are required for further development of this database as a necessary and important data source for modern selection practices in sheep.

There are two general ways of increasing the power of QTL detection—one by increasing the sample size of study cohorts and one by using a more powerful methodology for genetic analysis. The application of multivariate methods in genetic association studies can increase the statistical power to discover novel loci involved in the genetic control of quantitative traits [4,5,6]. Multivariate analysis of variance (MANOVA) is one of the most powerful multivariate methods for genetic association studies [6]. For certain traits, the application of MANOVA was empirically demonstrated to lead to non-trivial increases in the number of loci robustly identified in a replicable fashion [7]. Recently, this method has become applicable to the so-called GWAS summary statistics (full information about the effect sizes, effective and reference alleles, sample size, standard errors, and *p*-values for all available single nucleotide polymorphisms—SNPs). It should be noted that the application of MANOVA is a common practice in human genetics [8,9]. Its application in animal genetics is rapidly emerging [10].

In this study, we focused on the search for new loci associated with meat productivity and carcass traits in sheep using a modern multivariate approach. For the genome-wide association studies in humans, it is customary to provide summary statistics in the supplements to the published research, which is not the case for studies in sheep. However, there are exceptions. Bolormaa et al. [10] had previously provided full access to their summary-level GWAS data [10]. Using data from 10,613 animals, they identified 71 significant loci associated with 56 traits, in a multivariate joint analysis of all 56 traits using an approach similar to MANOVA. However, grouping traits by biological relationship and/or by correlation structure of the data allowed us to increase the power of genetic mapping and detect new loci [7]. In the present study, we exploited this approach to increase the number of loci associated with economically important traits and applied MANOVA to the summary statistics obtained in GWAS. We grouped 18 original univariate traits into three multivariate traits by biological relationship: the multivariate trait related to the general mass and mass of different parts (MMass), the multivariate trait that incorporates fat-related traits (MFat), and the multivariate trait that incorporates traits related to muscle properties (MMeat). Using multivariate analysis, we detected 12 associated loci, eight of which were reported for the first time. We replicated one of them in an independent sample of Russian sheep populations (*N* = 108).

## 2. Materials and Methods

### 2.1. Data Collection

We used the data on summary statistics (Z-scores) for 510,174 SNPs for 56 carcass composition traits provided by Bolormaa et al. (2016) [10]. The total sample size was 10,613. The sample consisted of nine breeds: Merino (MER), Poll Dorset (PD), Border Leicester (BL), Suffolk (SUF), white Suffolk (WS), Texel (TEX), Corriedale (CORR), Coopworth (COOP), and various Crosses (MIX). All animals were genotyped by Ovine Infinium^®^ HD SNP BeadChip, comprising 603,350 (HD) SNPs (Illumina Inc., San Diego, CA, USA) and the Illumina 50 k Ovine SNP chip (Illumina Inc., San Diego, CA, USA), comprising 54,241 (50 k) SNPs. Out of 10,613 animals with 50 k and 1735 HD genotypes, 1682 animals were genotyped by both SNP arrays. The imputation of the 50 k to HD was carried out using Fimpute [11]. In total, 10,613 animals had real or imputed HD genotypes for 510,174 autosomal SNPs and a phenotypic record for at least one trait. For more details, see [10].

### 2.2. Data Pre-Processing

We unified the original summary level data with the available Ovine Infinium^®^ HD SNP BeadChip reference (the Oar_v3.1 assembly) downloaded from [12]. SNPs with mismatches in chromosome position or rs number excluded. In total, 493,742 out of 510,174 SNPs passed quality control and were used in further analysis. The program code is freely available and can be found at https://github.com/Defrag1236/ovines_multivariate_analysis (access date: 3 March 2021).

### 2.3. Phenotype Grouping

Since this study is focused on the detection of loci associated with meat productivity, we used 18 out of 56 traits from the original study [10] directly related to mass, carcass, meat, and fat weights. We grouped these 18 traits in three multivariate traits by the similarity of their biological functions: the multivariate trait related to the general mass and the mass of different parts (MMass), the multivariate trait that incorporates fat-related traits (MFat), the multivariate trait that incorporates traits related to muscle properties (MMeat). The description of the groups is provided in Supplementary Table S1. The correlation matrix of all 56 traits is shown in Supplementary Figure S1. The correlation matrix for each respective multivariate trait is provided in Supplementary Figures S2–S4.

### 2.4. Multivariate GWAS Analysis

The multivariate analysis was performed using the summary statistics-based method described by Stephens (2013) [6] using the R environment [13]. This method enables the application of multivariate analysis of variance (MANOVA) using only the Z-scores (the ratio of effect size and its standard error) from the results of GWAS for several traits. In general, MANOVA is a generalized form of univariate analysis of variance (ANOVA) and uses the covariance between outcome variables in testing the statistical significance of the mean differences. In the context of genetic studies, MANOVA searches for the best linear combination of the original traits that is associated with a particular SNP. As a result, it gives the *p*-value of the association of each SNP with the group of the traits being studied. In our case, the MANOVA T-score for the *j*-th SNP was calculated as

$$T_{j}^{2}=Z_{j}^{|}\times V_{0}^{-1}\times Z_{j}$$

where $Z_{j}$ is the vector of Z-scores for the original traits and $V_{0}^{}$ is a matrix of phenotypic correlations. The matrix of phenotypic correlations was calculated as the correlation between Z-scores for the original traits using the approach proposed by Stephens (2013) [6]. The *p*-value was calculated using the R command pchisq T_score^2, df=X, low=F, where T_score is the T-score and X is the corresponding number of degrees of freedom (9 for MMass, 6 for MFat, and 3 for MMeat).

We used the threshold *p*-value $<\;0.05/\left(\mathrm{Nsnp}*3\right)$, where Nsnp = 493,742 is the number of SNPs that passed QC, and 3 is the number of multivariate traits.

The associated loci were defined as regions within ±500 kb around the SNPs most significantly associated with the trait (lead SNPs). We manually checked regional association plots for all loci found and merged two loci into one if they were located within the same linkage disequilibrium block. SNPs located between 22 Mb and 42 Mb on chromosome 6, between 24.5 Mb and 28 Mb on chromosome 11, and between 62 Mb and 67 Mb on chromosome 18 were merged into one locus each.

A locus was defined as new if it was not present in the list of known SNPs and genes from our previously published database of QTLs and genes associated with meat productivity in sheep [3]. This database contains information from 23 papers published between 2013 and 2020.

The proportion of variance explained by the locus and expressed as a percentage was calculated in the R environment as $\mathrm{qchisq}\left(p,\;\mathrm{df}=1,\;\mathrm{lower}.\mathrm{tail}=F\right)/10613)*100$, where *p* is the least *p*-value among those for 56 univariate traits.

Inflation factors for all multivariate traits and original univariate traits used for analysis are presented in Supplementary Table S2.

### 2.5. Regional Association Plots

Regional association plots were constructed using a homebrew script adjusted to the sheep genome annotation. The code is available in our Git repository (https://github.com/Defrag1236/ovines_multivariate_analysis, access date: 3 March 2021). We used Ensembl gene annotation (genome version OAR_3.1). The linkage disequilibrium (*r*^2^) between SNPs was calculated as the squared Pearson correlation between Z-scores for 56 original traits using the approach proposed by Stephens (2013) [6]. All regional association plots for discovered loci can be found in Supplementary Figures S8–S19.

### 2.6. Functional Annotation

We used the Ensembl variant effect predictor (VEP) to analyze the potential effects of SNPs and indels in high linkage disequilibrium (LD; *r*^2^ >  0.7) with the lead SNPs. The analysis was performed using software available online [14].

### 2.7. Literature-Based Prioritization

In order to detect promising candidate genes in associated loci, we performed a literature-based prioritization for genes located within 500 kb of the lead SNPs. We used the Uniprot database (http://www.uniprot.org, access date: 3 March 2021) to extract information about the short gene annotation, molecular function, and biological process of the gene. Due to the relative scarcity of data on sheep gene function, we supplemented our literature-based analysis with information published in the catalog of human genes Online Mendelian Inheritance in Man (OMIM) [15] and a mouse knockout database [16].

### 2.8. The Functional Network of Genes Associated with Meat Productivity Traits

The functional network is based on the assumption that the genes functionally connected and/or involved in one gene network will show a similar pattern of association with traits (in our case 56 traits). The original idea was presented in papers by Bolormaa et al. [10,17] and a recent study in human genetics [18]. To test our hypothesis, we used Spearman’s pairwise correlations calculated using original Z-scores. The sign of the correlation is irrelevant because the direction of the GWAS effect estimates depends on the allele used as a reference. Moreover, we named the loci after the prioritized genes rather than after rs IDs.

To construct the functional network, we performed a pairwise association analysis. Spearman’s correlation coefficient was computed for each pair from among 12 significantly associated SNPs [10,17,19]. As a threshold for the significance of Spearman’s coefficient, we used the *p*-value corrected for (12 × 11)/2 tests (*p*-value ≤ 7.6 × 10^−4^).

For the visualization of the functional network between genes with significant Z-based Spearman’s correlation, we used the “igraph” package for the R environment (https://www.r-project.org/, access date: 3 March 2021). Clustering and visualization were carried out using the “corrplot” package for R and the basic “hclust” function. For clustering, we estimated squared Euclidean distances by subtracting the absolute values of the genetic correlation from 1 and used Ward’s clustering method.

### 2.9. Replication in a Russian Independent Sample

The replication was performed on a mix of Russian sheep populations totaling 108 animals. The sample consisted of two genetic pools of animals. The first pool contained backcross hybrids from the Romanovskaya breed and argali (*Ovis ammon*). The second pool contained mixed hybrids of the Romanovskaya breed and Katahdin breed with argali and mouflon (*Ovis gmelini*).

All animals were genotyped by Ovine Infinium^®^ HD SNP BeadChip, comprising 603,350 (HD) SNPs (Illumina Inc., San Diego, CA, USA). The alleles were coded using the Illumina A/B forward/forward scheme. We filtered genotypes prior to analysis by MAF > 1% and by genotype call rate >95%. Next, we performed a PCA analysis of genotypes and manually checked whether the animals in each pool were genetically homogenous.

For all animals, phenotype information about 11 original traits was available for three-time intervals after the birth (6 days, 42 days, and 90 days). Based on these phenotypes, seven indexes (see Supplementary Table S3) were calculated for three-time intervals. Each of these indexes reflects the meat productivity, growth, and carcass properties of the animals. In total, we analyzed 24 traits (seven indexes and mass in three-time intervals) for replication analysis.

GWAS for each trait and for each pool of animals was performed using the GEMMA software [18], mixed-models, and the Wald test. The number of fetuses and sex were included as covariates. We used EMMAX [20] for the estimation of the genetic relatedness matrix using the Balding–Nichols method. For each phenotype and time interval, we meta-analyzed two pools of animals by inverse-variance weighted meta-analysis using the METAL software [21].

Next, we a performed multivariate analysis of three-time intervals on each of the eight phenotypes using the approach described above. Only traits with a univariate sample size of more than 20 were included in the multivariate analysis. In total, we obtained eight multivariate GWAS with a sample size varying from 50 to 108. Inflation factors for each of the univariate and multivariate traits are presented in Supplementary Table S4. As additional validation, we checked the association of four known loci. Locus rs401834107 was associated with width slope index at *p* < 1.106 × 10^−5^, which is below the Bonferroni corrected threshold (*p*-value$\;<0.05/\left(8\times 12\right)$). The other three loci had a minimum *p*-value higher than the threshold.

Only six out of eight novel loci were analyzed. Locus rs403766990 was excluded because of a low MAF (<1%), and locus rs428034699 was excluded because of a low genotypic call rate (<95%). The threshold for replication was set to $0.05/\left(6\times 8\right)=0.001$, where 6 is the number of novel SNPs for replication and 8 is the number of multivariate traits in the replication cohort. Moreover, if SNP was significant at least for one of the multivariate traits we have checked the consistency between the direction of the meat productivity effects estimated for the traits with univariate *p*-value by Bolorma et al. [10] and in our replication study.

## 3. Results

### 3.1. Multivariate GWAS Analysis

**Figure 1:**
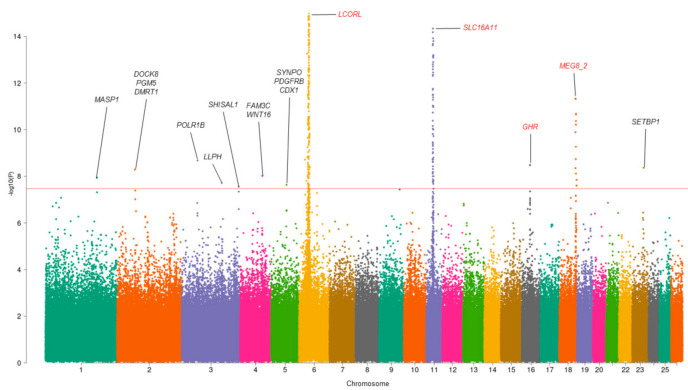
Manhattan plot for MANOVA for three multivariate traits. For each SNP, the minimum *p*-value among three multivariate traits is indicated. For each significant locus, the prioritized genes are presented. Known loci are marked by red. SNPs with *p*-values < 1 × 10^−14^ are omitted. *MASP1*: mannan-binding lectin serine peptidase 1; *DOCK8*: dedicator of cytokinesis 8; PGM5: phosphoglucomutase 5; *DMRT1*: doublesex and mab−3 related transcription factor 1; *POLR1B*: RNA polymerase I subunit B; *LLPH*: LLP homolog, long-term synaptic facilitation factor; *SHISAL1*: shisa like 1; *FAM3C*: FAM3 metabolism-regulating signaling molecule C; *WNT16*: Wnt family member 16; *SYNPO:* synaptopodin; *PDGFRB*: platelet-derived growth factor receptor β; *CDX1*: caudal type homeobox 1; *LCORL*: ligand-dependent nuclear receptor corepressor like; *SLC16A11*: solute carrier family 16 member 11; *GHR*: growth hormone receptor; *MEG8_2:* maternally expressed 8, small nucleolar RNA host gene; *SETBP1*: SET binding protein 1.

The results of the MANOVA analysis for three multivariate traits are presented in Table 1 (for detailed results, see Supplementary Table S5). We found nine loci significantly associated with MMass and three loci significantly associated with MFat (*p*-value < 3.38× 10^−8^). The strongest association was demonstrated for three known loci on chromosomes 6, 11, and 18 (the *LCORL1, SLCA11*, and *MEG8_2* genes, respectively). The Manhattan plot for MANOVA GWAS results is presented in Figure 1. The quantile-quantile (Q-Q) plots are presented in Supplementary Figures S5–S7. It should be noted that there is a deviation of the QQ plot from the middle line. In the original study, Bolormaa et al. [10] demonstrated the same behavior of the QQ plot for the original 56 traits. In our opinion, this is a result of the highly polygenic nature of the traits and a very large sample size rather than confounding. Four out of twelve loci had been previously reported to be associated with different ovine traits [3,10].

### 3.2. Literature-Based Gene Prioritization

The summary of an in silico follow-up for eight novel loci is presented in Supplementary Table S6. First, we performed VEP (the results are given in Supplementary Table S7). We detected one missense mutation (rs193632759, MET > ILE) located in the *MASP1* gene. This gene is involved in the lectin complement pathway that facilitates the recognition of pathogens through oligosaccharide chains and their subsequent elimination [22].

Details of the literature-based annotation are given in Supplementary Table S8. Three independent researchers made a decision about which gene or genes could be potentially prioritized in each region. In total, we have prioritized 13 genes for 8 loci (details are given in Supplementary Table S8).

For the locus tagged by rs420734786, we were not able to prioritize one specific gene. The lead SNP is positioned in the *DOCK8* gene, which is involved in immune reactions. Defects in this gene in humans result in combined immunodeficiency [23]. However, in close proximity to this SNP, two genes are located—*PGM5*, which plays a role in muscle development and can be a potential candidate for further studies [24], and *DMRT1*, which is involved in sex determination and has a systemic effect on the development of the carcass [25].

In the locus tagged by rs401990068, we focused on two genes—*FAM3C* and *WNT16*. *FAM3C* is the nearest gene to the lead SNP in this locus. This gene may be involved in the retinal laminar formation and play a role in multicellular organism development. *WNT16* is involved in the Wnt/CTNNB1 signaling pathway and may play redundant roles in synovial joint induction [26].

On chromosome 5, in the locus tagged by the rs408893215, we prioritized three genes. *SYNPO* is the nearest gene to the lead SNP and plays a role in actin binding. *PDGFRB* encodes platelet-derived growth factor receptor β. In humans, this gene has been proposed by several authors as a therapeutic target [27,28,29]. Moreover, this gene may play a role in growth factor binding. *CDX1* was shown to be expressed in the colon and intestine of adult humans [30].

For the loci tagged by rs399851221, rs428034699, rs403766990, and rs418394153, we proposed the nearest genes (*POLR1B, LLPH, SETBP1, SHISAL1*) as candidates. *POLR1B* plays a role in bone structure development [31]. The *SETBP1* gene deficiency can cause the Schinzel–Giedion syndrome in humans. One of the symptoms is the stagnation of growth in children. Products of this gene are also associated with bone and connective tissue cancer cells and implicitly involved in bone development [32].

### 3.3. The Functional Network of Loci Associated with Meat Productivity Traits in Sheep

**Figure 2:**
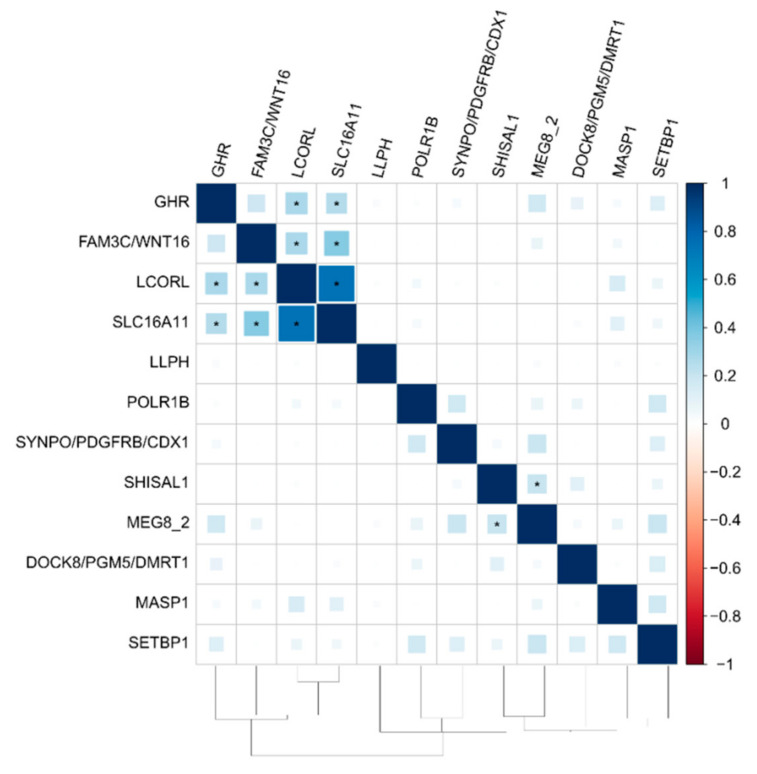
Heatmap of Spearman’s pairwise locus-effect correlations. Each significant correlation is asterisked (*p*-value ≤ 7.6 × 10^−4^). Color intensity depends on the correlation value. *MASP1*: mannan-binding lectin serine peptidase 1; *DOCK8*: dedicator of cytokinesis 8; *PGM5*: phosphoglucomutase 5; *DMRT1*: doublesex and mab−3 related transcription factor 1; *POLR1B*: RNA polymerase I subunit B; *LLPH*: LLP homolog, long-term synaptic facilitation factor; *SHISAL1*: shisa like 1; *FAM3C*: FAM3 metabolism-regulating signaling molecule C; *WNT16*: Wnt family member 16; *SYNPO*: synaptopodin; *PDGFRB*: platelet-derived growth factor receptor β; *CDX1*: caudal type homeobox 1; *LCORL*: ligand-dependent nuclear receptor corepressor like; *SLC16A11*: solute carrier family 16 member 11; *GHR*:; *MEG8_2*: maternally expressed 8, small nucleolar RNA host gene; *SETBP1*: SET binding protein 1.

**Figure 3:**
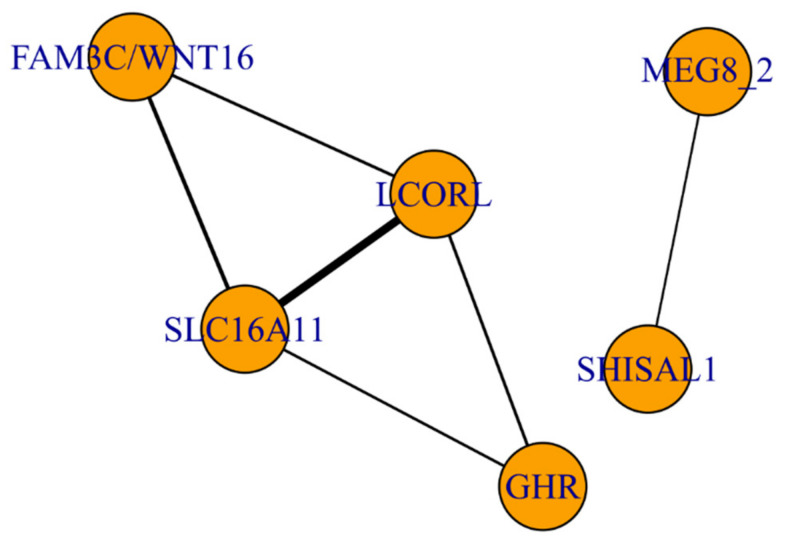
The functional network of loci associated with meat productivity traits. Each node represents a lead SNP in the locus and each edge represents squared Spearman’s pairwise locus-effect correlation (*p*-value ≤ 7.6 × 10^−4^). *FAM3C*: FAM3 metabolism-regulating signaling molecule C; *WNT16*: Wnt family member 16; *SLC16A11*: solute carrier family 16 member 11; *GHR*: growth hormone receptor; MEG8_2: maternally expressed 8 small nucleolar RNA host gene, SHISAL1: shisa like 1.

The heatmap of Spearman’s correlation matrix is presented in Figure 2. The functional graph for the genes with at least one significant Spearman’s correlation (*p*-value ≤ 7.6E-04) is presented in Figure 3.

The strongest overall correlation (*r* = 0.74) was observed between two known loci on chromosome 6 (*LCORL*) and chromosome 11 (*SLC16A11*). In addition, these loci are connected with the *FAM3C/WNT16* and GHR genes. Together, these loci form a clear functional subnetwork (Figure 3). Another known locus on chromosome 18 (*MEG8_2*) was connected with a novel locus on chromosome 3 (the *SHISAL1* gene) (Figure 3).

### 3.4. Replication in the Russian Independent Sample

We replicated novel loci using a mix of Russian sheep populations (*N* = 108). We performed a multivariate analysis of eight traits related to meat productivity and carcass. Six out of eight loci were subjected to replication. Locus rs401990068 near *FAM3C* and *WNT16* was significantly associated (*p*-value < 1.106 × 10^−5^) with long-legged index (see Supplementary Table S3). *P*-values for all 10 loci (six new and four previously known) for each of the eight multivariate traits are presented in Supplementary Table S9. For rs401990068, we confirmed the consistency between the alleles that increase meat productivity in the Russian cohort and the original study by Bolormaa et al. [10] and considered this locus replicated.

## 4. Discussion

Our results demonstrate that it is possible to extract additional useful information from published data through the use of different methods. In our opinion, reporting full summary-level data may greatly accelerate the accumulation of relevant information about SNPs and their association with economically important traits in sheep. This practice is widely adopted in human GWAS studies.

Interestingly, five of eight novel loci were associated with MMass, the multivariate trait representing general body mass. The number of novel loci associated with the traits representing fat or muscle composition was only three. Among 56 original traits, we have detected the ones most associated with a lead SNP for each new locus (see Supplementary Table S5). For three loci, the most associated univariate trait was leg bone weight. Dressing and hot carcass weight were among the most significant univariate traits. Thus, we may speculate that the genes located in the new loci are likely to alter the carcass and bone properties, potentially increasing the animal size and, consequently, increasing the animal mass.

We performed gene prioritization for the novel loci. For the locus tagged by rs193632759, we found a missense mutation in the MASP1 gene, which gives us relatively high confidence that this gene is functional for the association. For the locus tagged by rs425579441, there was only one annotated gene (*SETBP1*) within the 500-kb region (±500 kb away from the lead SNP), making this gene the only candidate for the locus. For the other loci, our confidence in the prioritized genes is not well supported due to the use of literature-based prioritization only. For six loci, our prioritization was based on cumulative evidence from the literature and on the physical proximity of the gene. In total, we have prioritized 13 genes for 8 novel loci. Additional studies are needed to find out what gene is truly functional in the loci with ambiguous prioritization.

We also replicated the novel loci in the Russian sheep population. Although the settings of the cohort were not optimal for replication and the sample size was not large enough to guarantee the optimum power, we replicated one of six novel loci (rs401990068) included in the replication study. Another locus near *DOCK8/PGM5/DMRT1* was close to the threshold (*p*-value = 0.0014) but was not considered as replicated. Further replication studies are needed with larger sample sizes to confirm the association of the other novel loci.

It should be noted that for 3 loci out of the 12 discovered, we observed large blocks of linkage disequilibrium (between 22 Mb and 42 Mb on chromosome 6, between 24.5 Mb and 28 Mb on chromosome 11, and between 62 Mb and 7 Mb on chromosome 18). Those loci are highly associated with meat and carcass traits (the proportion of variance explained for the most associated univariate trait was approximately 3.42%, 1.42%, and 0.48% for loci on chromosomes 6, 11, and 18, respectively). It is most likely that these loci contain a haplotype or several haplotypes with a particularly strong effect and should consequently be considered as some of the most important loci for practical use and selection. A more detailed study of these regions would require a different methodology for fine mapping and/or sequencing of the regions. Moreover, we have shown that these loci together with the novel locus *FAM3C/WNT16* are parts of the same functional network (see Figure 3). Given that the *LCORL* gene is a known transcription factor and the strongest association signal for meat productivity traits, we may speculate that LCORL directly regulates the expression of genes in the other three loci, which most probably consist of receptors, enzymes, and ion channels.

Finally, we have uploaded information about eight new loci into a recently developed database of QTLs and genes associated with growth and meat traits in sheep [3]. In total, the database now contains more than 130 unique loci. This information can be useful for further association studies and preliminary estimation of genetic variability in different breeds.

## 5. Conclusions

Eight novel loci containing the *MASP1, DOCK8/PGM5/DMRT1, POlR1B, LLPH, SHISAL1, FAM3C/WNT16, SYNPO/PDGFRB/CDX1,* and *SETBP1* genes are associated with mass, fat, and muscle composition traits in sheep. The loci with the *FAM3C/WNT16, LCORL, SLC16A11,* and *GHR* genes are parts of one functional network with shared regulation. Association of locus rs401990068 was replicated on an independent sample of Russian sheep populations.

## Figures and Tables

**Table 1:** The 12 loci significantly associated with at least one multivariate trait.

SNP	CHR	POS	*p*-Value_MMass	*p*-Value_MFat	*p*-Value_MMeat	ra/ea	Gene
rs193632759 *	1	198273462	**1.14 × 10** ^**−8**^	0.95	0.11	T/G	*MASP1*
rs420734786 *	2	68158297	**5.12 × 10** ^**−9**^	0.56	0.74	A/C	*DOCK8* *PGM5* *DMRT1*
rs399851221 *	3	60513720	**2.08 × 10** ^**−9**^	0.30	0.38	T/C	*POLR1B*
rs403766990 *	3	153924034	0.02	**1.93 × 10** ^**−8**^	0.05	A/G	*LLPH*
rs428034699 *	3	219082890	**2.78 × 10** ^**−9**^	0.25	0.41	T/C	*SHISAL1*
rs401990068 *	4	85985834	0.41	**9.62 × 10** ^**−9**^	0.90	T/G	*FAM3C* *WNT16*
rs408893215 *	5	59475661	0.06	**2.31 × 10** ^**−8**^	0.01	T/C	*SYNPO* *PDGFRB* *CDX1*
rs401834107	6	37530647	**6.04 × 10** ^**−94**^	9.73 × 10^−24^	5.11× 10^−12^	T/C	*LCORL*
rs161042491	11	26445930	**2.34 × 10** ^**−41**^	2.64 × 10^−10^	1.15 × 10^−6^	A/G	*SLC16A11*
rs405660596	16	31871071	**3.28 × 10** ^**−9**^	0.03	8.73 × 10^−4^	A/C	*GHR*
rs408838557	18	62894338	**4.64 × 10** ^**−12**^	5.04 × 10^−8^	1.29 × 10^−3^	A/C	*MEG8_2*
rs418394153 *	23	44492468	**4.27 × 10** ^**−9**^	0.60	0.28	A/G	*SETBP1*

Asterisk denotes novel loci. Trait: the most significantly associated trait; Traits: all traits significantly associated with the locus; CHR: the chromosome; POS: the position of the SNP on the chromosome (according to the OAR 3.1 assembly); *p*-value_MMass, *p*-value_MFat, *p*-value_MMeat: *p*-values for the corresponding traits; ra/ea: reference and effective alleles; Gene: prioritized genes. The most significant *p*-value for each locus is in bold.

## Data Availability

The full results of multivariate GWAS are available from the authors upon reasonable request.

## Supplementary Materials

The following are available online at https://www.mdpi.com/2073-4425/12/3/367/s1, Table S1: Trait grouping for multivariate analysis, Table S2: Inflation factors for univariate and multivariate traits, Table S3: Definition of each index from the replication cohort, Table S4: Inflation factors for univariate and multivariate traits in the Russian independent sample, Table S5: Detailed results of MANOVA analysis, Table S6: Summary of the in silico follow-up, Table S7: Results of the variant effective predictor (VEP), Table S8. Details of literature-based gene prioritization, Table S9: *P*-value for 21 traits in the replication cohort, Figure S1: Correlation matrix for 56 original traits from a study by Bolormaa et al. (2016) [10]; Figure S2: Correlation matrix for 9 original traits from MMass multivariate trait, Figure S3: Correlation matrix for six original traits from MFat multivariate trait, Figure S4: Correlation matrix for three original traits from MMeat multivariate trait, Figure S5: QQ plot for MMass multivariate trait, Figure S6: QQ plot for MFat multivariate trait, Figure S7: QQ plot for MMeat multivariate trait, Figure S8: Regional association plot for locus rs193632759, Figure S9: Regional association plot for locus rs420734786, Figure S10: Regional association plot for locus rs399851221, Figure S11: Regional association plot for locus rs403766990, Figure S12: Regional association plot for locus rs428034699, Figure S13: Regional association plot for locus rs401990068, Figure S14: Regional association plot for locus rs408893215, Figure S15: Regional association plot for locus rs401834107, Figure S16: Regional association plot for locus rs161042491, Figure S17: Regional association plot for locus rs405660596, Figure S18: Regional association plot for locus rs408838557, Figure S19: Regional association plot for locus rs418394153.
