# Supplementary figures and images for: Multivariate Analysis Identifies Eight Novel Loci Associated with Meat Productivity Traits in Sheep
Source: Genes (Basel). 2021 Mar 4;12(3):367. doi: 10.3390/genes12030367 (PMC8002146; doi:10.3390/genes12030367)

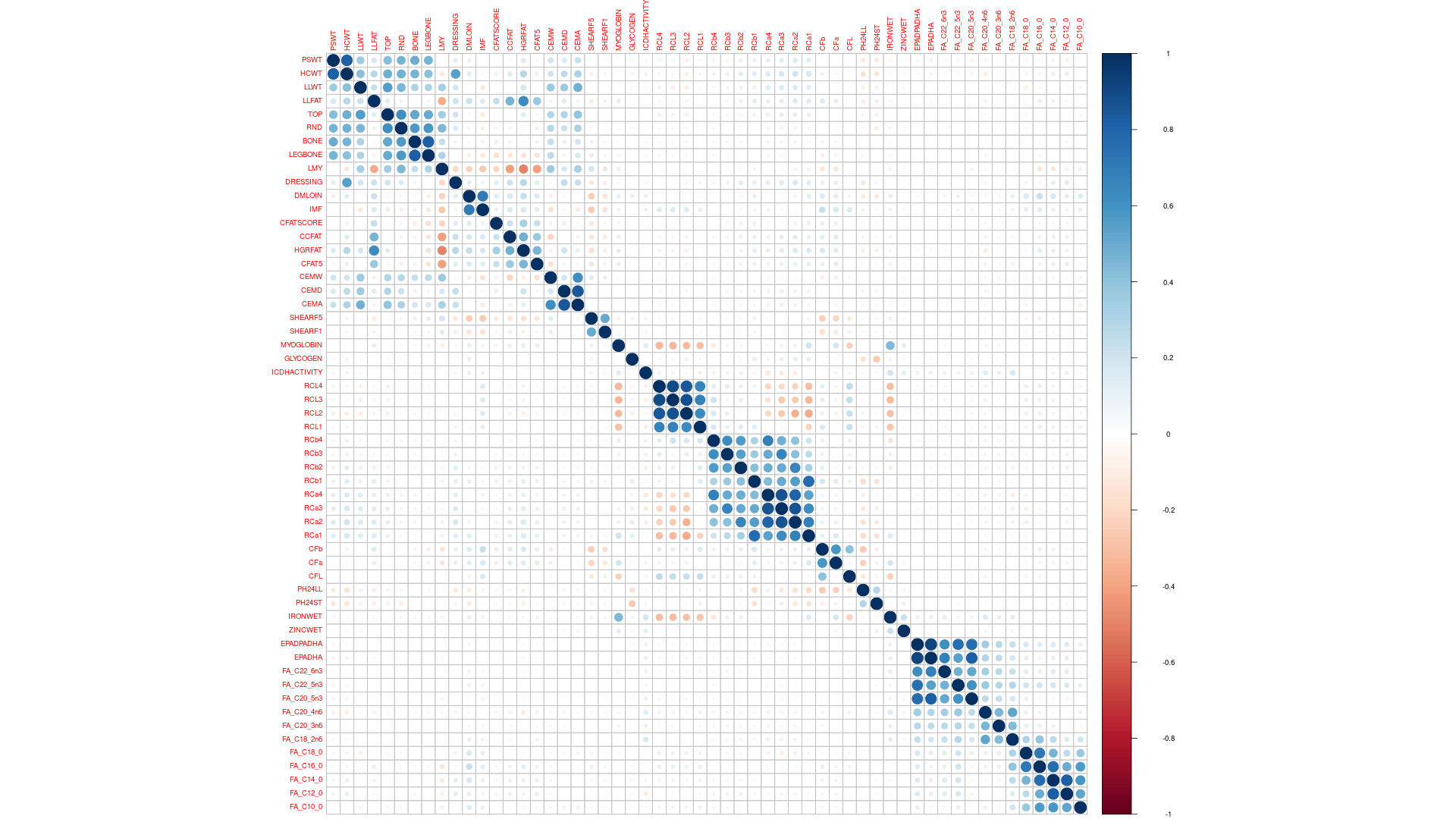

Supplement: Supplementary file 1 [file genes-12-00367-s001.zip › supplementary/supplementary/clear_supplementary_figures_for paper/Supplementary_figure_1.png]

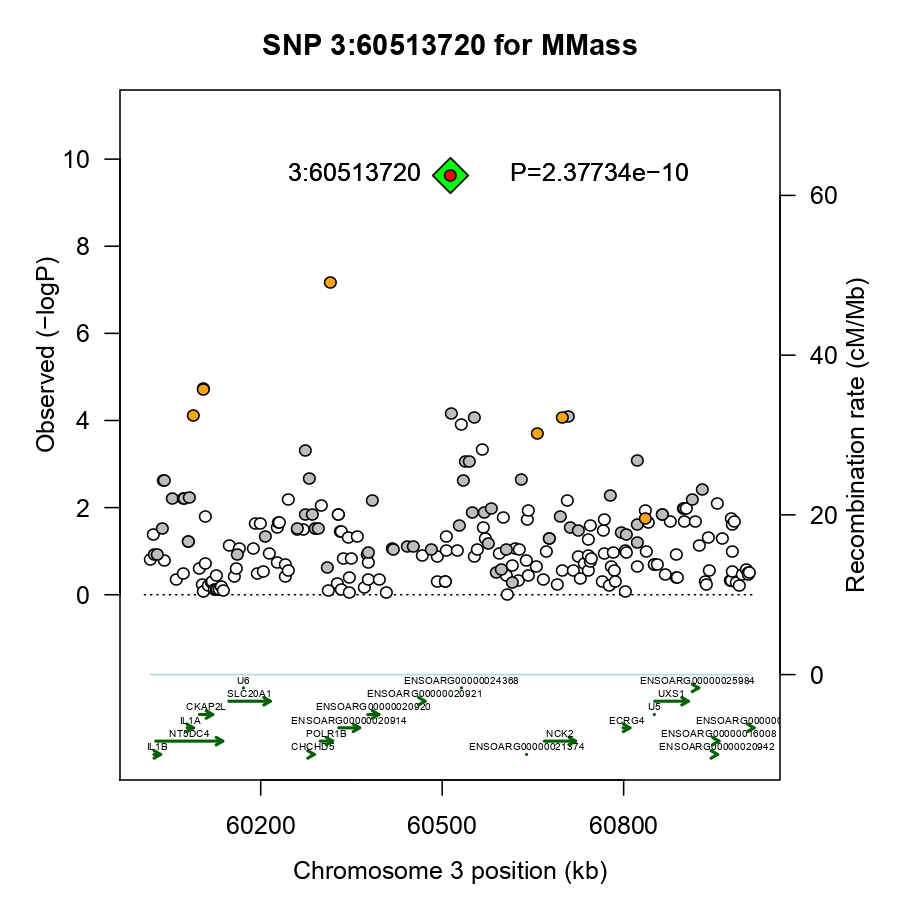

Supplement: Supplementary file 1 [file genes-12-00367-s001.zip › supplementary/supplementary/clear_supplementary_figures_for paper/Supplementary_figure_10.jpg]

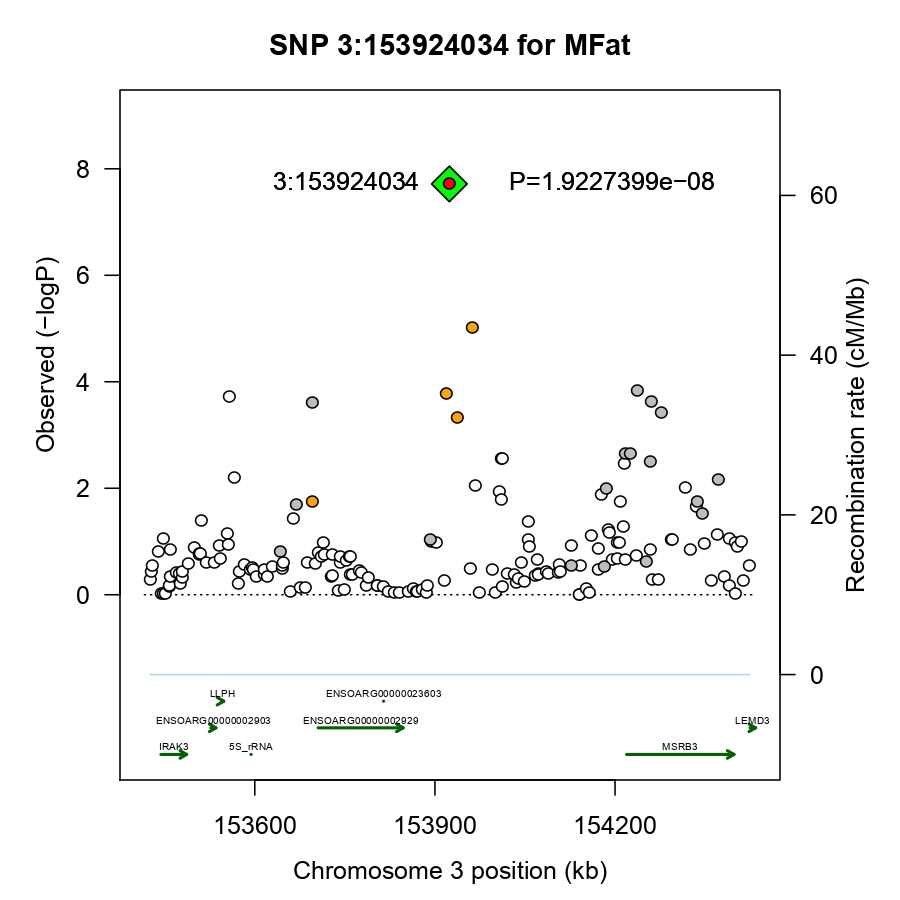

Supplement: Supplementary file 1 [file genes-12-00367-s001.zip › supplementary/supplementary/clear_supplementary_figures_for paper/Supplementary_figure_11.jpg]

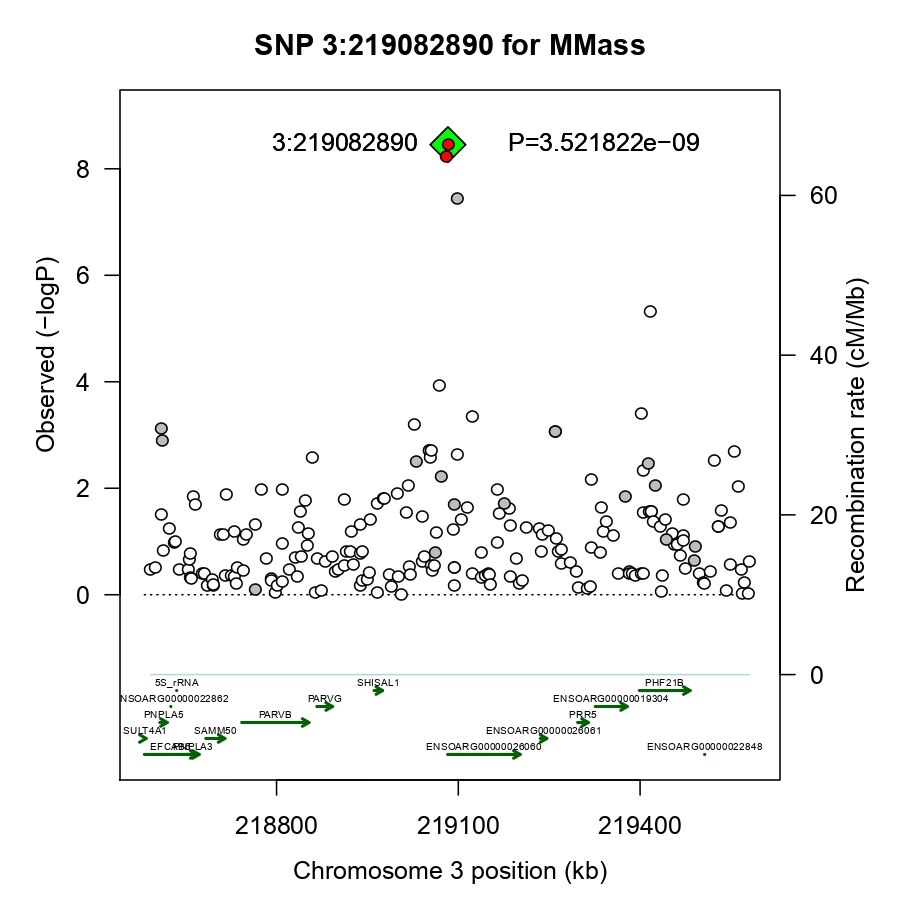

Supplement: Supplementary file 1 [file genes-12-00367-s001.zip › supplementary/supplementary/clear_supplementary_figures_for paper/Supplementary_figure_12.jpg]

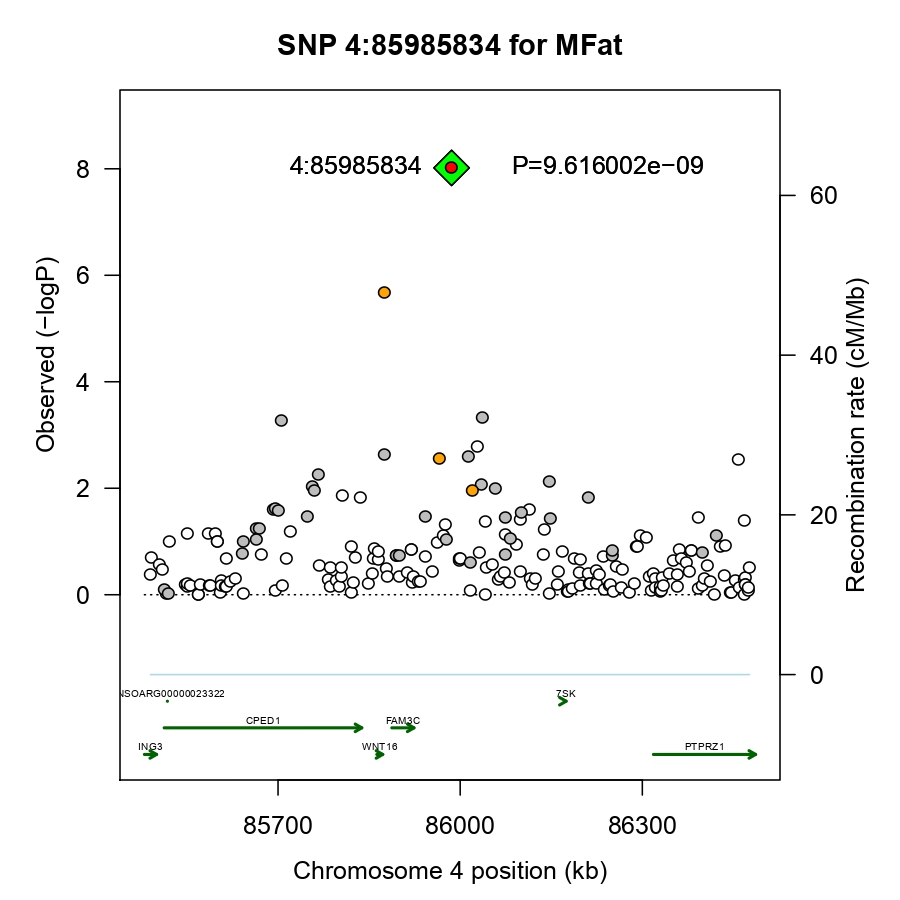

Supplement: Supplementary file 1 [file genes-12-00367-s001.zip › supplementary/supplementary/clear_supplementary_figures_for paper/Supplementary_figure_13.jpg]

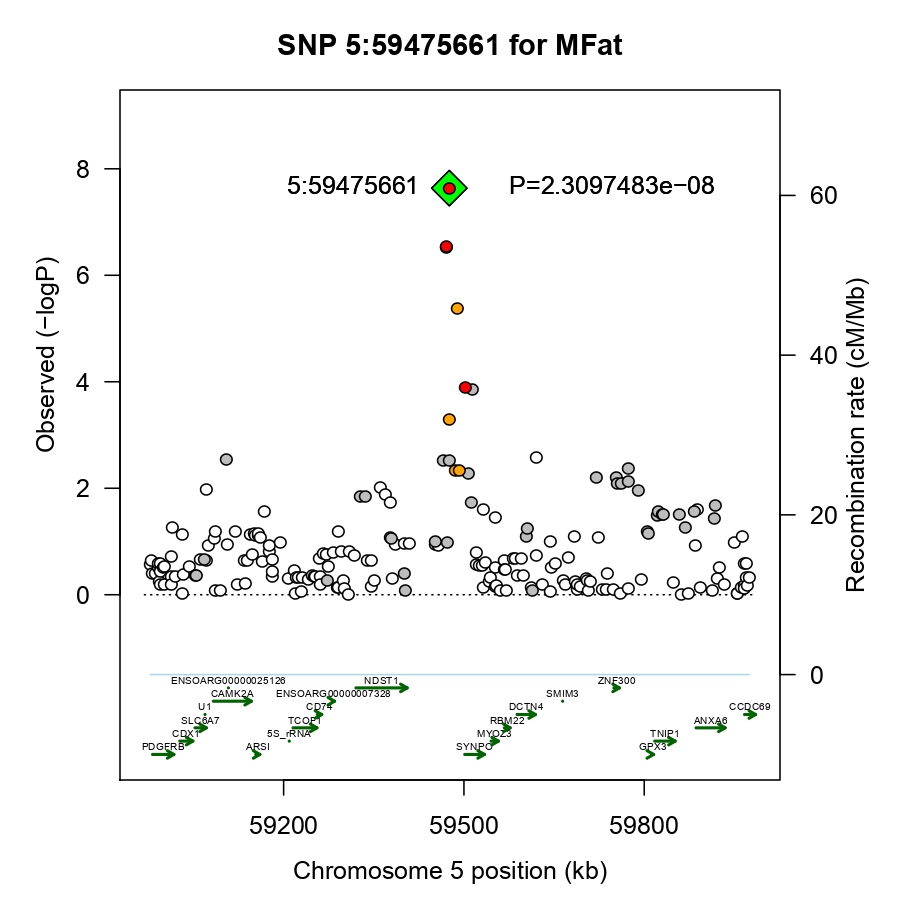

Supplement: Supplementary file 1 [file genes-12-00367-s001.zip › supplementary/supplementary/clear_supplementary_figures_for paper/Supplementary_figure_14.jpg]

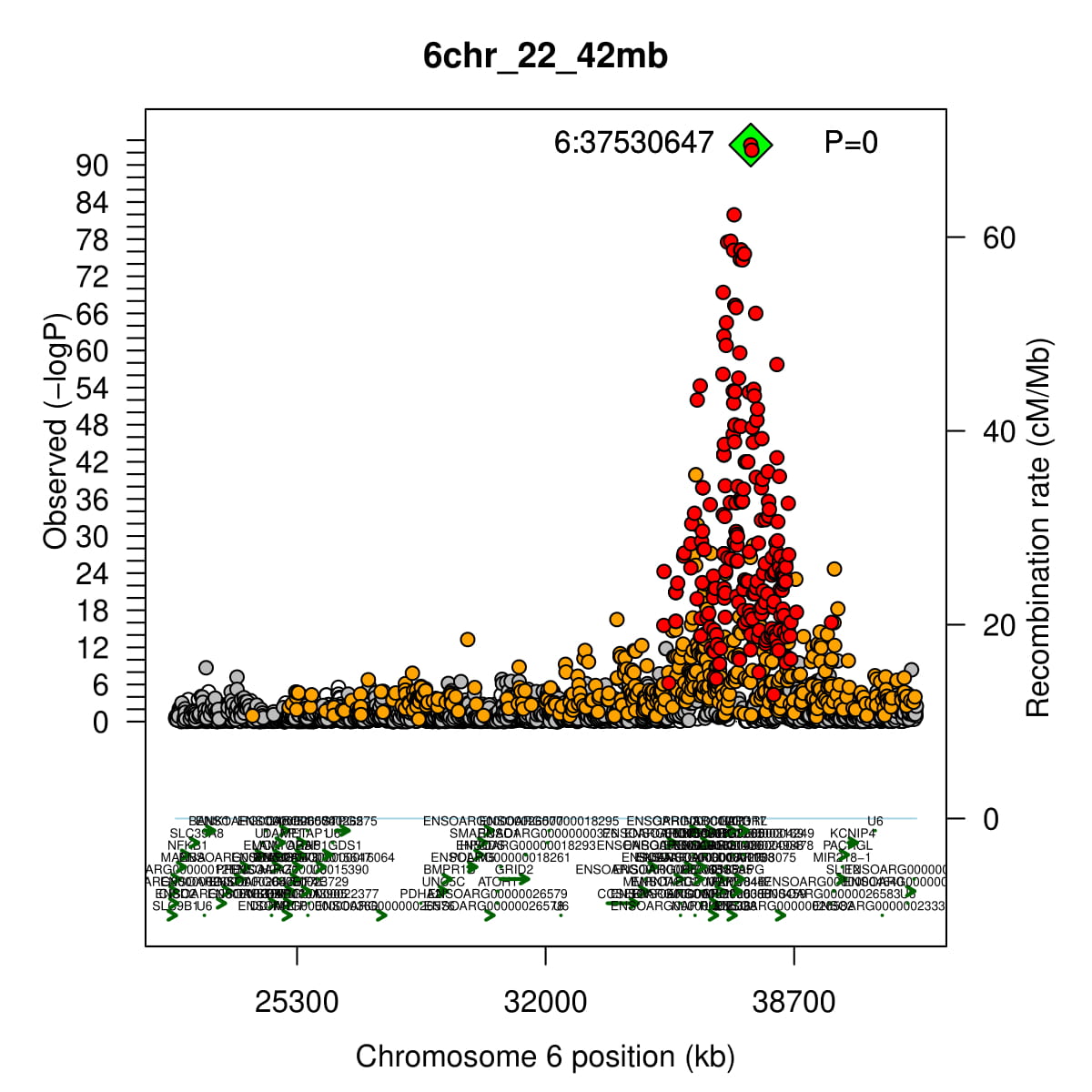

Supplement: Supplementary file 1 [file genes-12-00367-s001.zip › supplementary/supplementary/clear_supplementary_figures_for paper/Supplementary_figure_15.jpg]

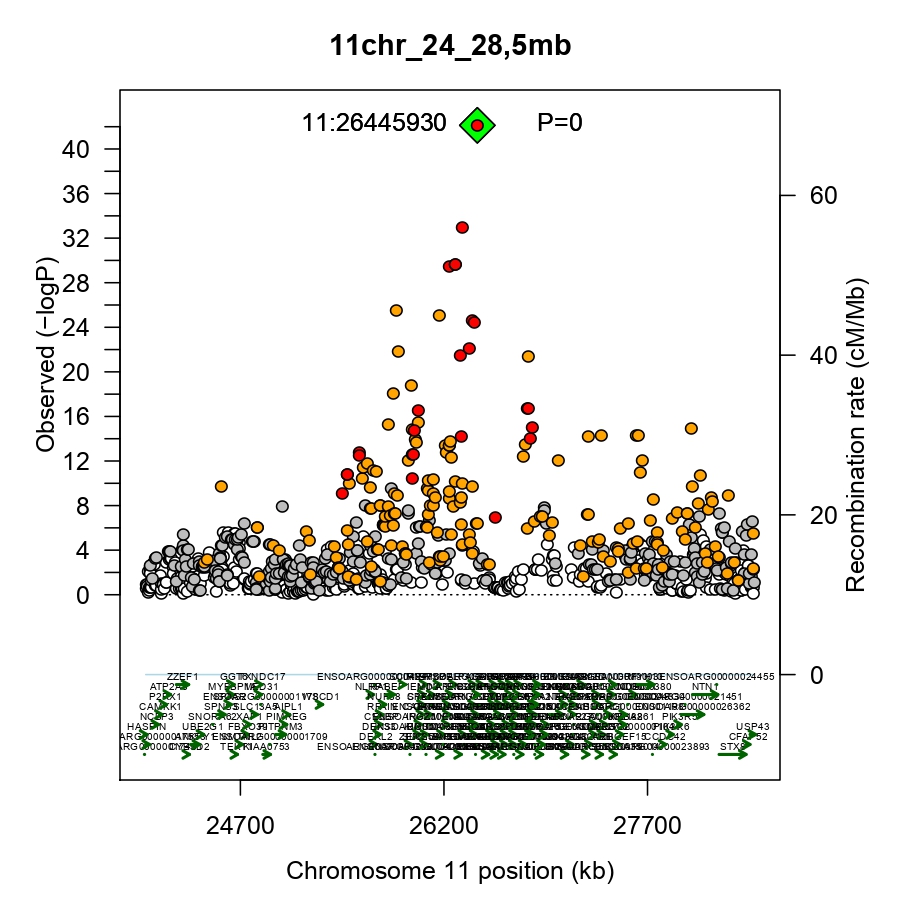

Supplement: Supplementary file 1 [file genes-12-00367-s001.zip › supplementary/supplementary/clear_supplementary_figures_for paper/Supplementary_figure_16.jpg]

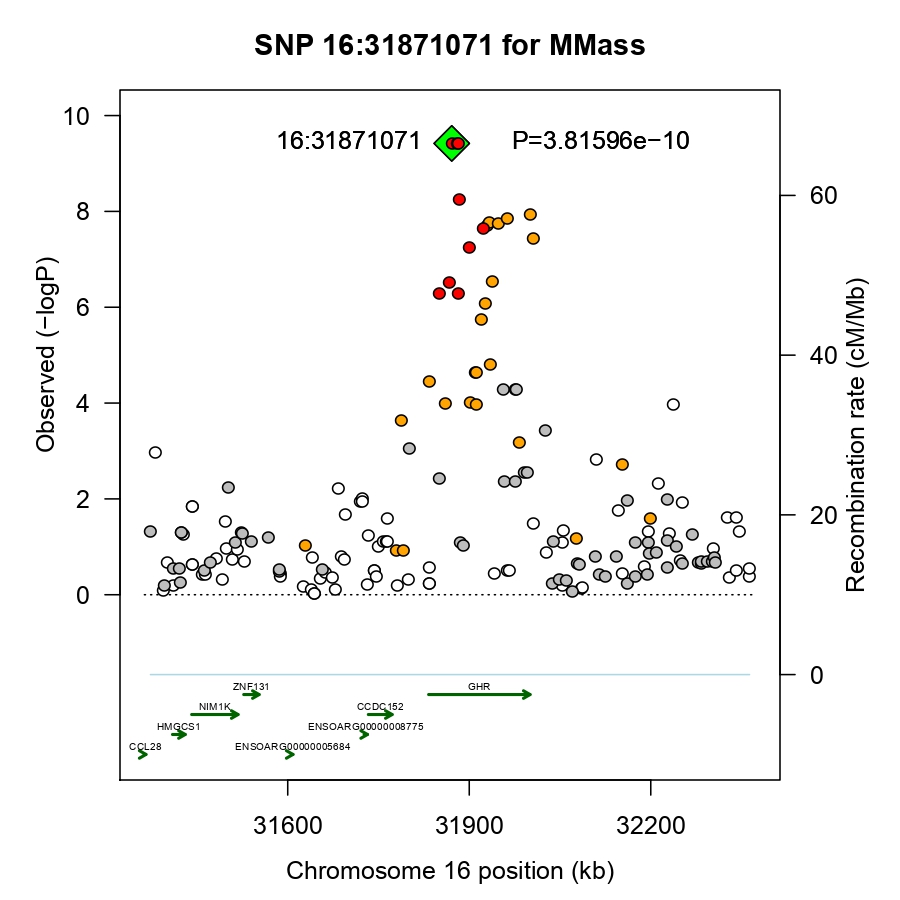

Supplement: Supplementary file 1 [file genes-12-00367-s001.zip › supplementary/supplementary/clear_supplementary_figures_for paper/Supplementary_figure_17.jpg]

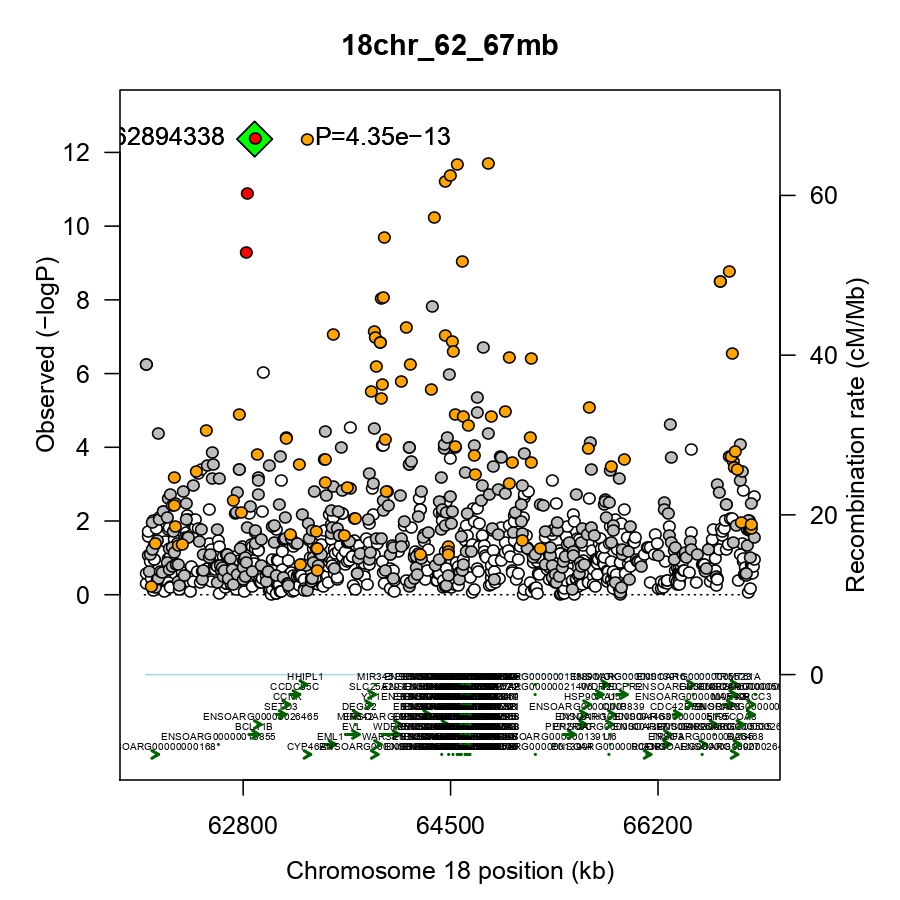

Supplement: Supplementary file 1 [file genes-12-00367-s001.zip › supplementary/supplementary/clear_supplementary_figures_for paper/Supplementary_figure_18.jpg]

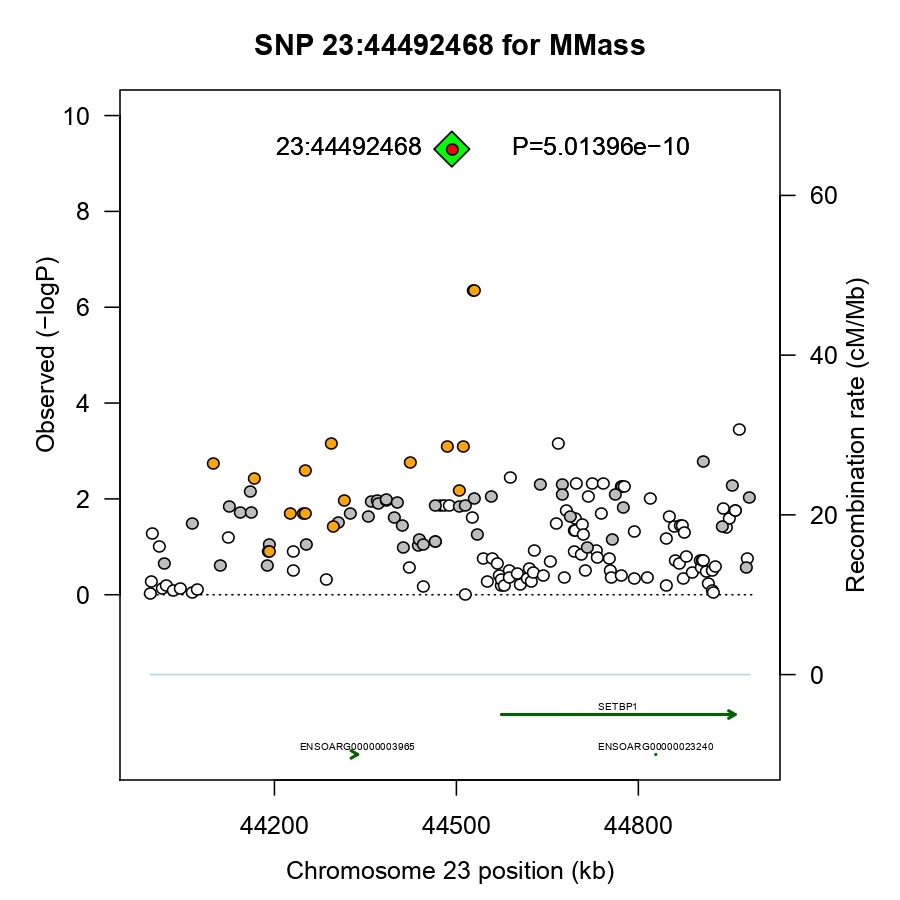

Supplement: Supplementary file 1 [file genes-12-00367-s001.zip › supplementary/supplementary/clear_supplementary_figures_for paper/Supplementary_figure_19.jpg]

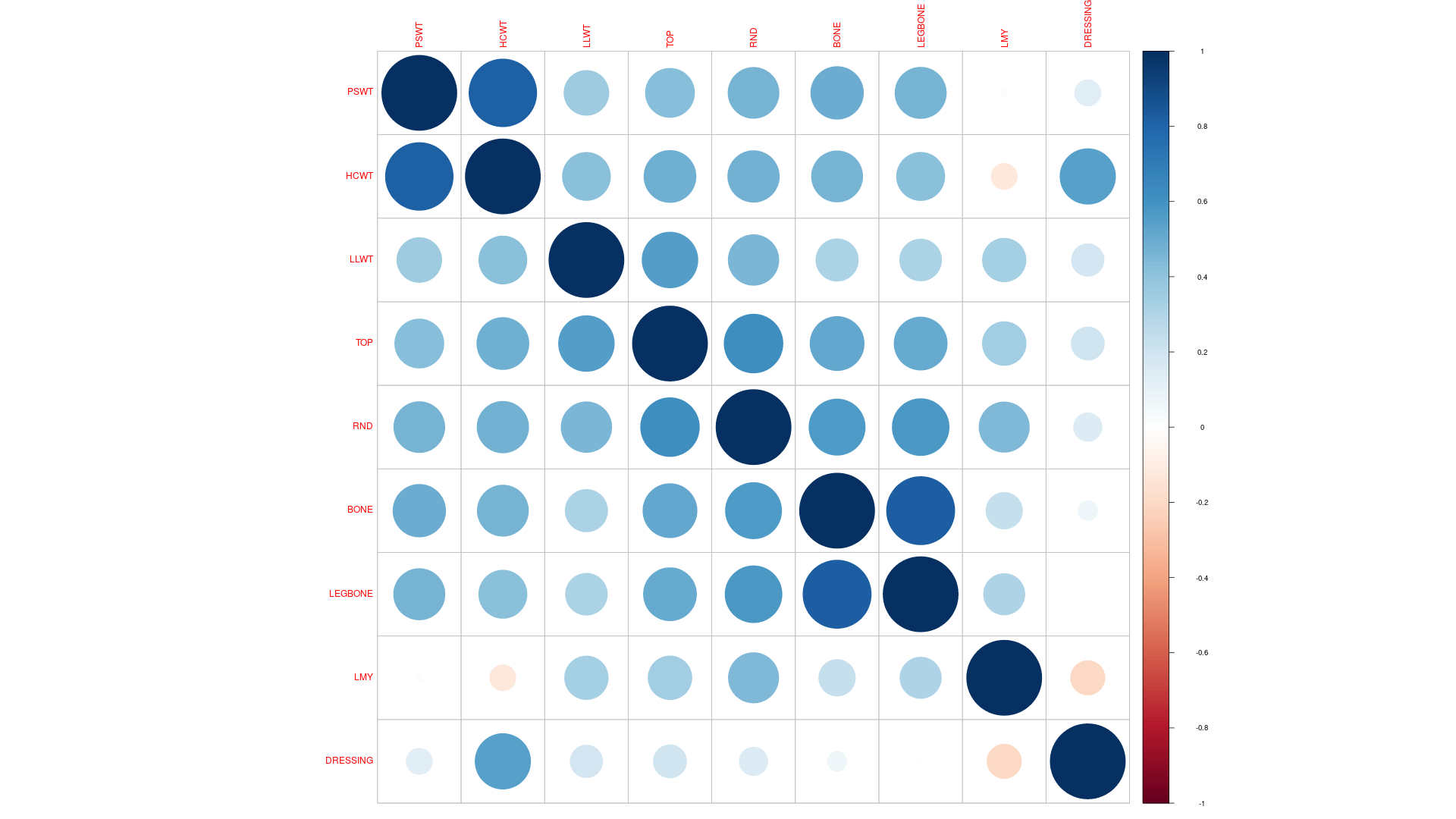

Supplement: Supplementary file 1 [file genes-12-00367-s001.zip › supplementary/supplementary/clear_supplementary_figures_for paper/Supplementary_figure_2.png]

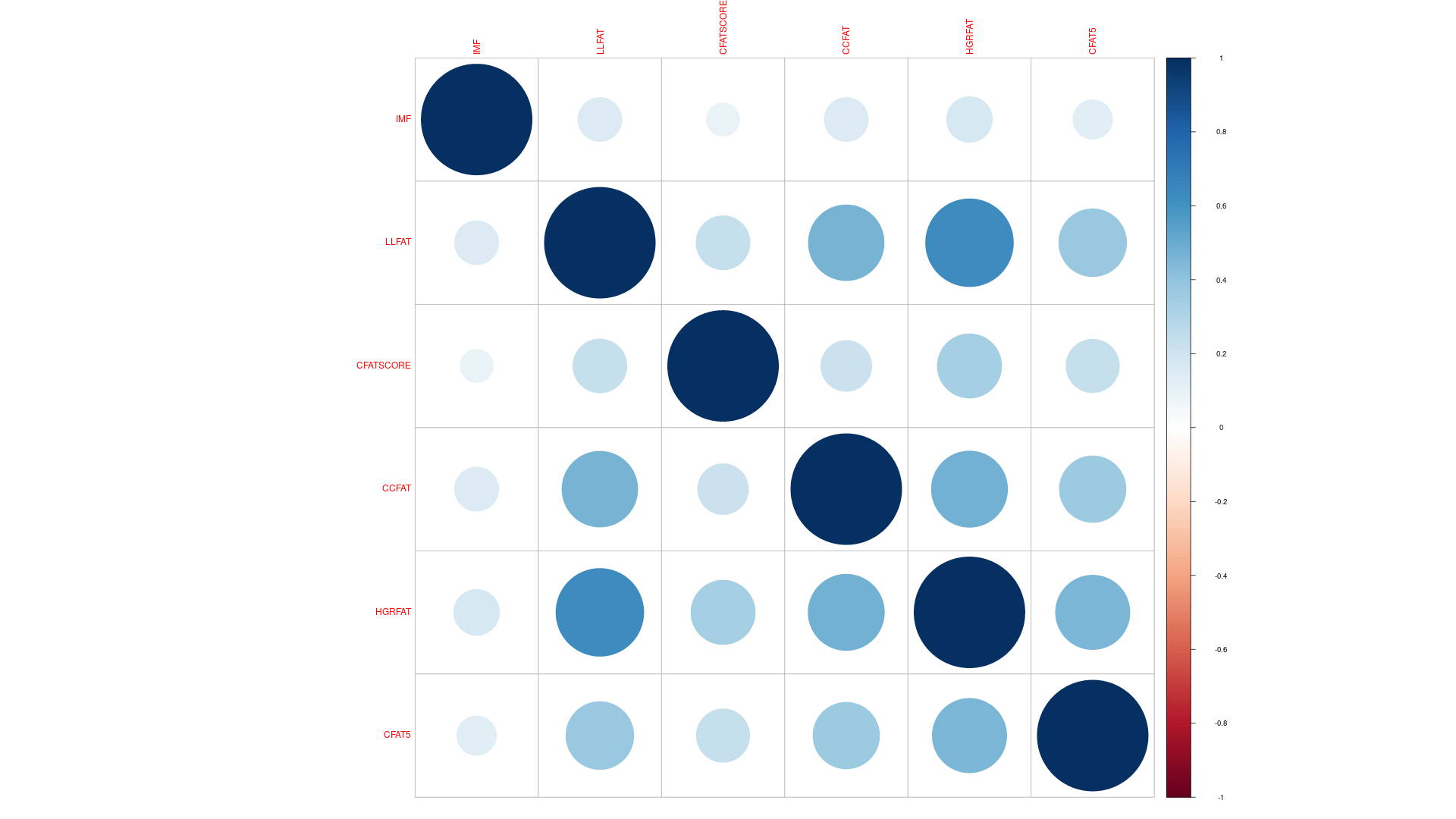

Supplement: Supplementary file 1 [file genes-12-00367-s001.zip › supplementary/supplementary/clear_supplementary_figures_for paper/Supplementary_figure_3.png]

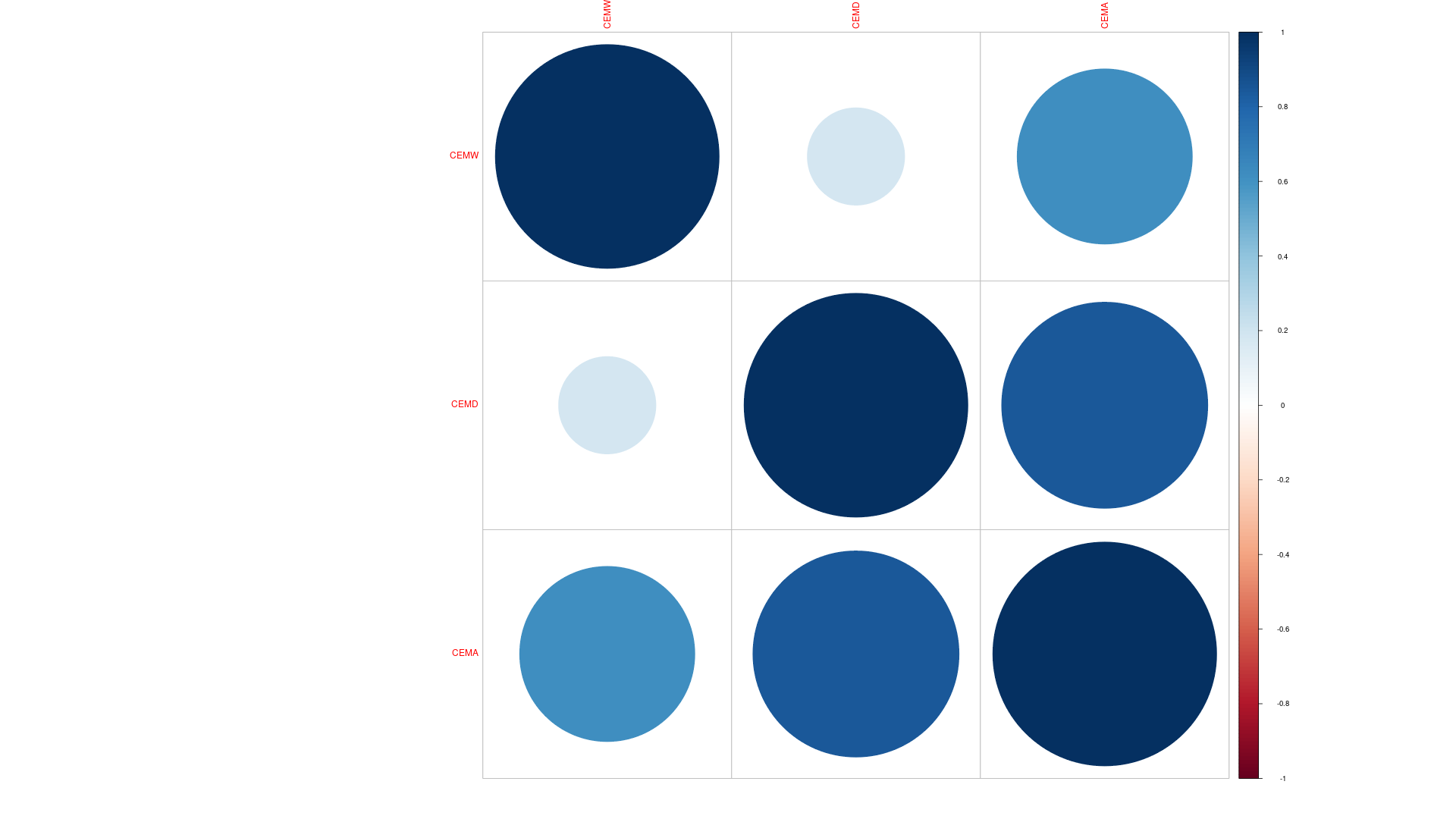

Supplement: Supplementary file 1 [file genes-12-00367-s001.zip › supplementary/supplementary/clear_supplementary_figures_for paper/Supplementary_figure_4.png]

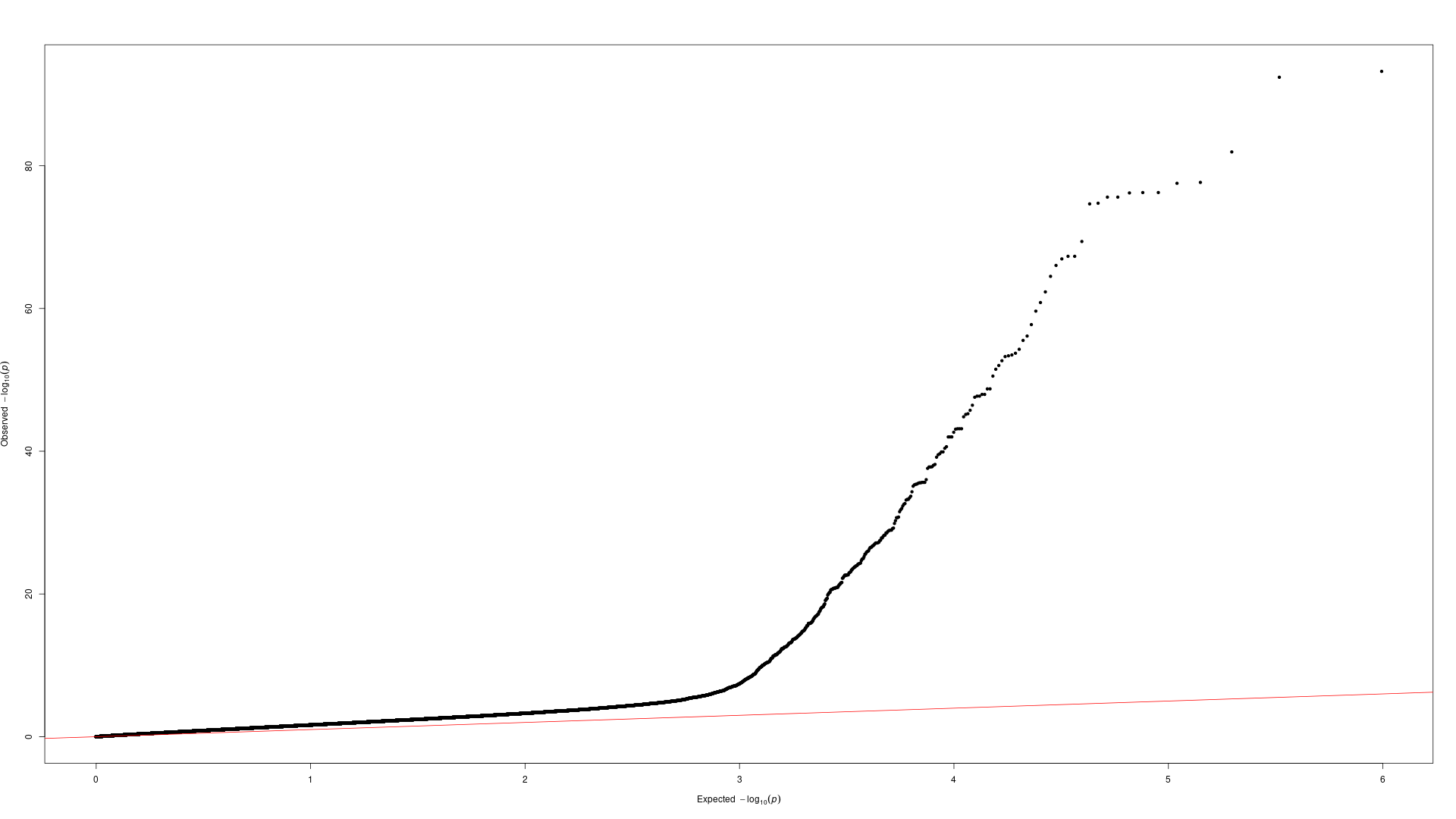

Supplement: Supplementary file 1 [file genes-12-00367-s001.zip › supplementary/supplementary/clear_supplementary_figures_for paper/Supplementary_figure_5.png]

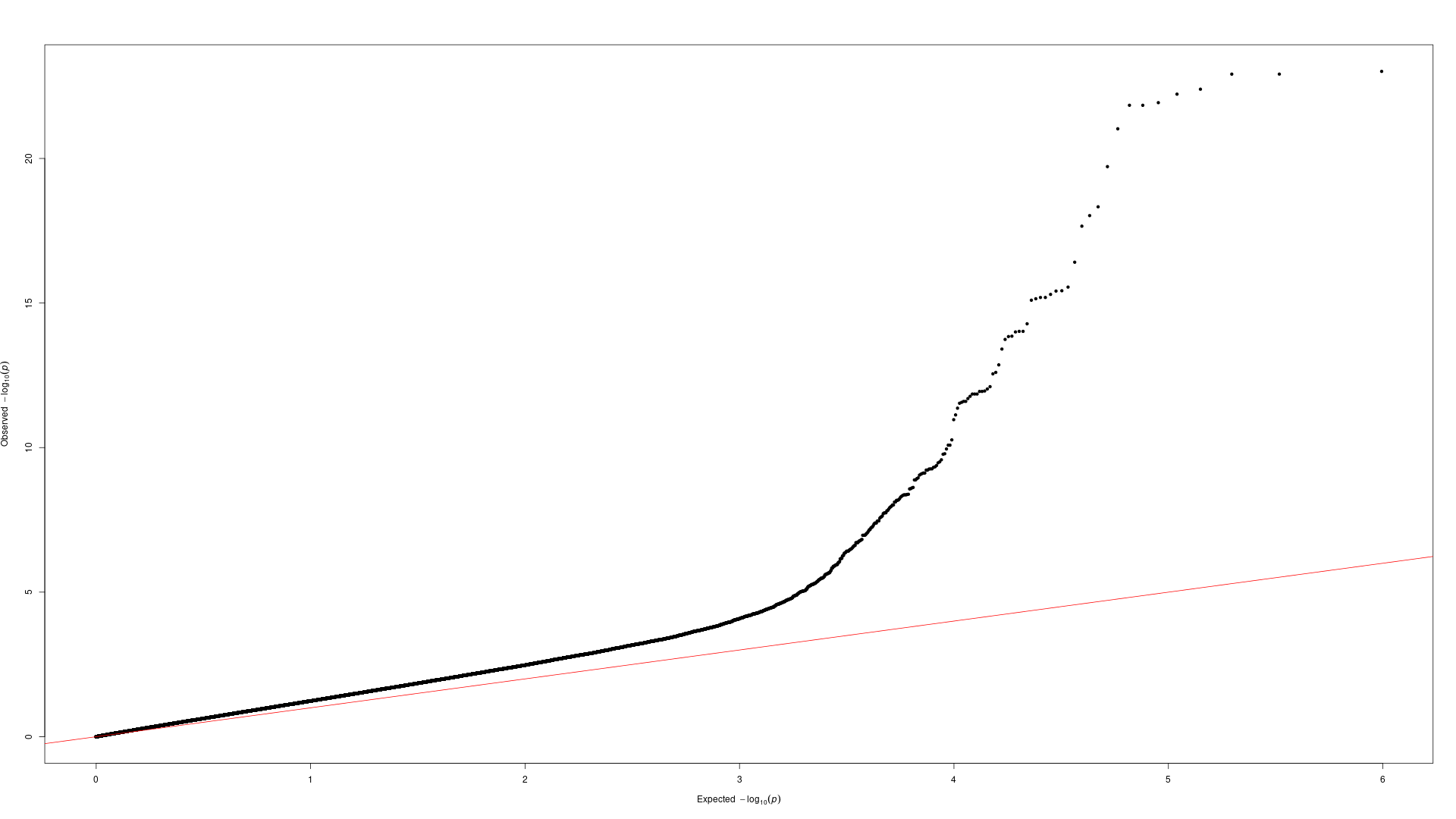

Supplement: Supplementary file 1 [file genes-12-00367-s001.zip › supplementary/supplementary/clear_supplementary_figures_for paper/Supplementary_figure_6.png]

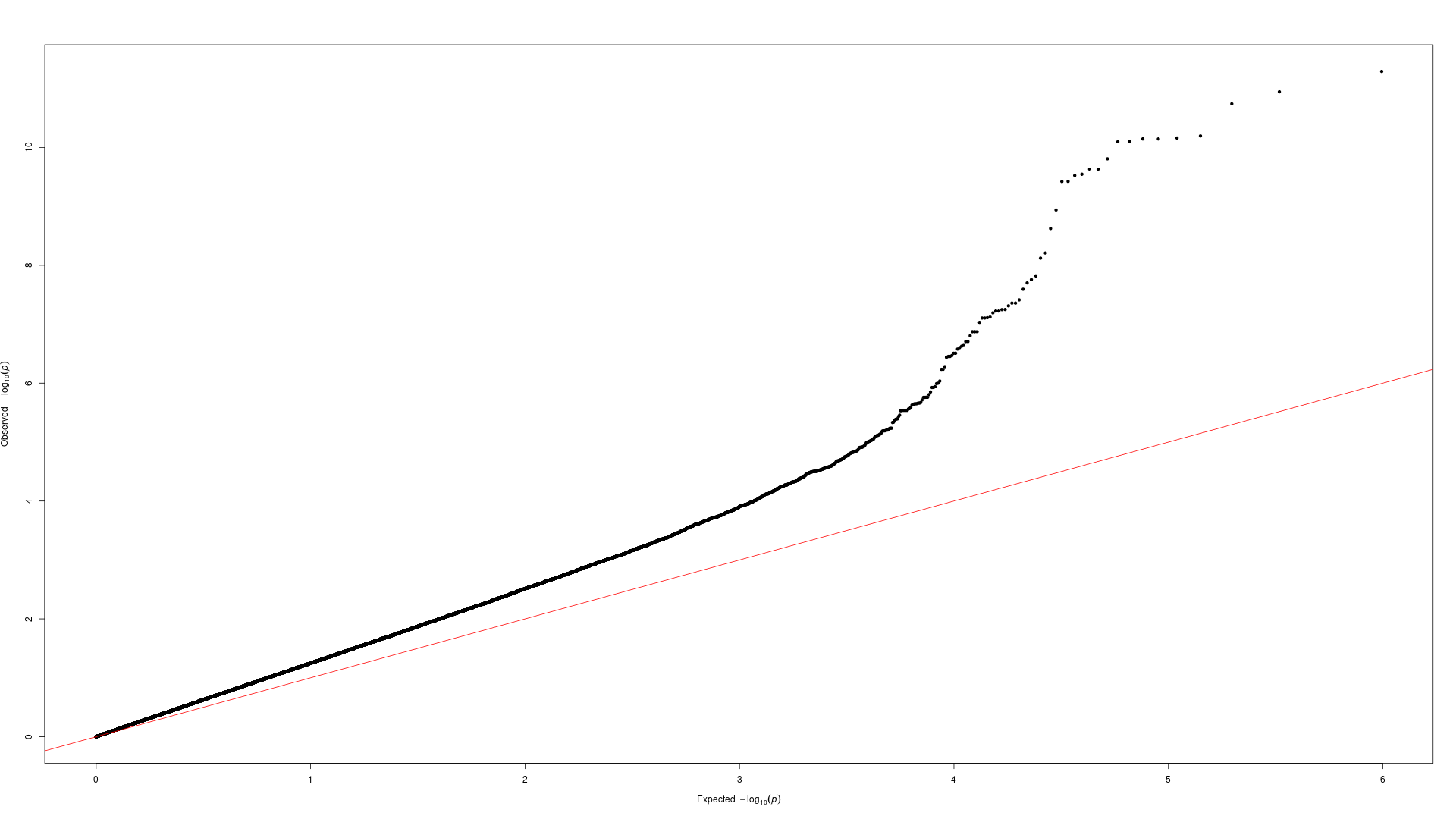

Supplement: Supplementary file 1 [file genes-12-00367-s001.zip › supplementary/supplementary/clear_supplementary_figures_for paper/Supplementary_figure_7.png]

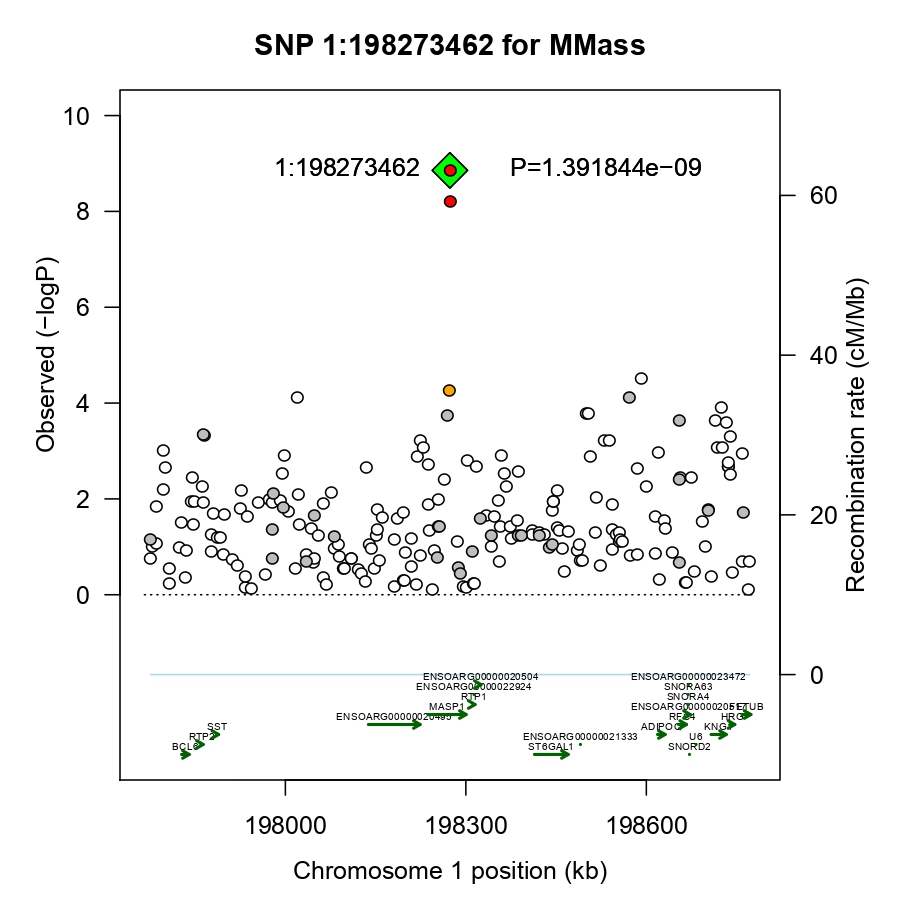

Supplement: Supplementary file 1 [file genes-12-00367-s001.zip › supplementary/supplementary/clear_supplementary_figures_for paper/Supplementary_figure_8.jpg]

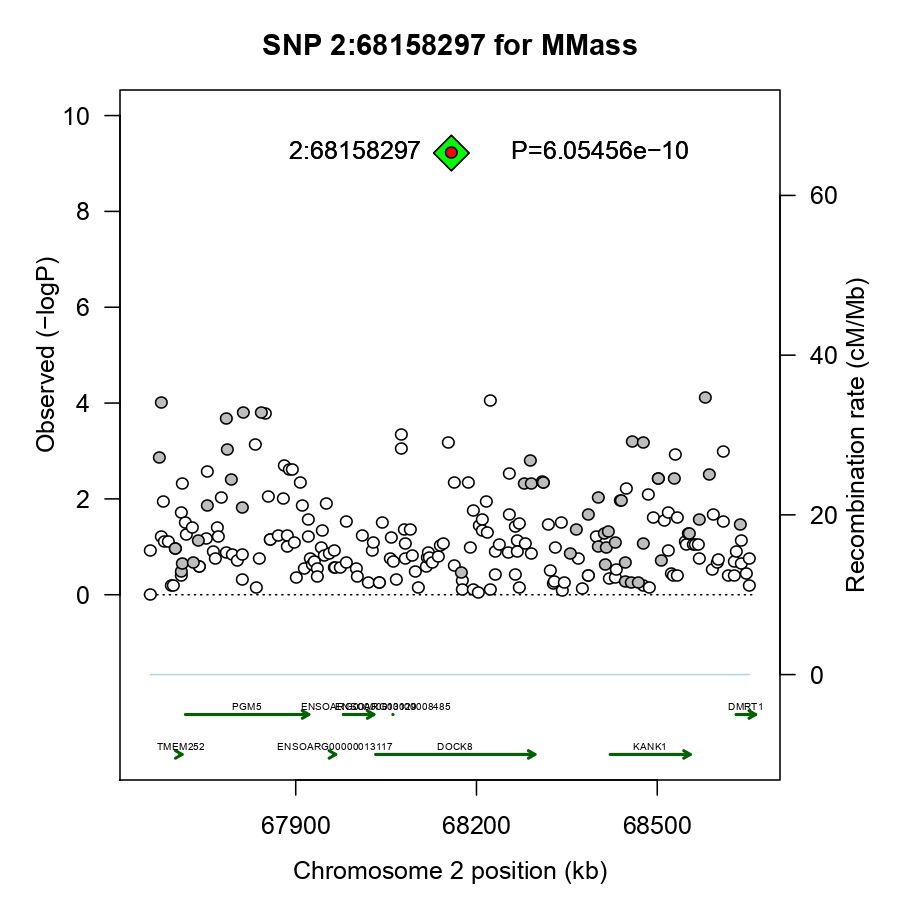

Supplement: Supplementary file 1 [file genes-12-00367-s001.zip › supplementary/supplementary/clear_supplementary_figures_for paper/Supplementary_figure_9.jpg]
